# Speech deficits in multiple sclerosis: a narrative review of the existing literature

**DOI:** 10.1186/s40001-023-01230-3

**Published:** 2023-07-24

**Authors:** Panagiotis Plotas, Vasiliki Nanousi, Anastasios Kantanis, Eirini Tsiamaki, Angelos Papadopoulos, Angeliki Tsapara, Aggeliki Glyka, Efraimia Mani, Fay Roumelioti, Georgia Strataki, Georgia Fragkou, Konstantina Mavreli, Natalia Ziouli, Nikolaos Trimmis

**Affiliations:** 1 Department of Speech and Language Therapy, School of Health Rehabilitation Sciences, University of Patras, Patras, Greece; 2 Laboratory of Primary Health Care, School of Health Rehabilitation Sciences, University of Patras, Patras, Greece; 3 Department of Neurology, Medical School, University of Patras, Patras, Greece

**Keywords:** Multiple sclerosis, Speech deficits, Phonation system, Articulation system, Prosody, Dysarthria, Dysphonia

## Abstract

Multiple sclerosis (MS) is a chronic inflammatory and demyelinating autoimmune disease. MS patients deal with motor and sensory impairments, visual disabilities, cognitive disorders, and speech and language deficits. The study aimed to record, enhance, update, and delve into our present comprehension of speech deficits observed in patients with MS and the methodology (assessment tools) studies followed. The method used was a search of the literature through the databases for May 2015 until June 2022. The reviewed studies offer insight into speech impairments most exhibited by MS patients. Patients with MS face numerous communication changes concerning the phonation system (changes observed concerning speech rate, long pause duration) and lower volume. Moreover, the articulation system was affected by the lack of muscle synchronization and inaccurate pronunciations, mainly of vowels. Finally, there are changes regarding prosody (MS patients exhibited monotonous speech). Findings indicated that MS patients experience communication changes across various domains. Based on the reviewed studies, we concluded that the speech system of MS patients is impaired to some extent, and the patients face many changes that impact their conversational ability and the production of slower and inaccurate speech. These changes can affect MS patients’ quality of life.

## Introduction

Multiple sclerosis (MS) is a chronic, progressive, autoimmune disease of the central nervous system, ‘characterized by inflammation, demyelination, followed by neurodegeneration’ [1]. MS is caused by damage to the myelin sheath, i.e., the protective covering of nerve fibers (axons) disrupting in that way the transmission of the nerve impulses to and from the central nervous system leading to specific clinical symptoms [2]. It also damages the nerve cell bodies (and their axons) in the brain, spinal cord and optic nerves affecting the transmission of visual information from the eye to the brain [3]. MS has traditionally been characterized as a persistent inflammatory ailment affecting the central nervous system [4]. This results in significant focal lesions in the white matter of the brain and spinal cord, marked by primary demyelination and varying degrees of axonal loss [4]. The presence of a dense glial scar in long-standing established lesions is associated with a profound astroglia reaction in the brain of individuals with MS, which in turn is linked to demyelination and neurodegeneration [4–6]. As the disease progresses the cerebral cortex shrinks leading to cortical atrophy [5, 6]. In addition, neuronal impairment has the potential to impact various bodily functions such as vision, sensation, coordination, movement, and bladder or bowel control [7]. This can lead to a range of neurogenic lower urinary tract symptoms in individuals with multiple sclerosis (MS), which have been reported to significantly impact their quality of life [8, 9]. MS can be diagnosed at any age; it most commonly manifests itself between ages 20 to 40, while the average age of onset is 30 years [10]. MS affects women twice as much as men [11]. MS etiology is still unclear, but it seems to be a disorder with great heterogeneity both among patients as well as within the same patient [12]; it is multifactorial attributed to both environmental and genetic factors [13].

There is a great variety of signs and symptoms in MS and many patterns have been identified: benign; a relapsing–remitting course; a secondary progressive type; and a primary progressive type [14]. In MS patients, the initial neurological signs and symptoms are subclinical, lasting for at least 24 h. This clinical presentation is known as clinically isolated syndrome (CIS) [14]. MS patients exhibit a range of symptoms reflecting multifocal lesions within the central nervous system affecting the motor, sensory, and visual systems. Thus, MS has a great impact on quality of life, as patients suffer from fatigue and mental difficulties [15], emotional distress [16], including depression, anxiety, negative mood, and trauma symptoms [17], alongside the sensory and motor limitations. Impairment of the motor system can subsequently affect the quality of communication [18].

The literature indicated that a large proportion of the MS population is impaired on standard neuropsychological tests, including verbal skills [18, 19]. Regarding the neuroanatomical pathophysiology of MS, cortical and subcortical brain structures have been identified to play a crucial role in the adjustment and coordination of the movement aspects of speech [20]. Communication is disrupted by the occurrence of motor speech disorders (dysarthria) that potentially affects all speech subsystems including, respiration, phonation, resonance, articulation, and prosody, along with impairments in receptive and expressive language [21]. Dysarthria is a prevalent motor speech disorder observed in individuals with MS, which restricts their communicative capacity in social situations and consequently impacts their overall quality of life [22, 23]. According to existing literature [24], individuals with MS may exhibit three distinct types of dysarthria, namely spastic dysarthria, ataxic dysarthria, and mixed dysarthria. Unique patterns of speech symptoms characterize these types of dysarthria. Nevertheless, it is worth noting that dysarthria is not the sole speech impairment that can be observed in the speech of individuals with multiple sclerosis. [24]. In addition, in MS, the lack of voluntary coordination of muscle movements is referred to as ataxia, which can cause speech problems [25]. Many difficulties concerning other domains such as voice, fluency and rate of speech are also noted which in turn have a significant effect on patients’ everyday communication [26]. The crucial responsibility of speech and language pathologists is to not only recognize and tackle speech impairments, but also to assist individuals in engaging in their daily routines by acquiring and implementing compensatory techniques to mitigate these challenges and enhance their overall well-being. Moreover, a recent meta-analysis has indicated that respiratory muscle training can enhance lung volumes and respiratory muscle strength in neuromuscular conditions such as multiple sclerosis (MS)[27].

### Assessment tools

Given that the preliminary indications of the ailment often become apparent during the initial stages of adulthood, it is imperative to conduct neurological and neuropsychological assessments at an early stage of the diagnostic procedure for individuals who have been diagnosed with MS or are suspected to have MS. In this field, comprehensive neuropsychological test like the Minimal Assessment of Cognitive Function in MS [28], the Brief International Cognitive Assessment for MS [29] and the shortened version of Rao's Brief Repeatable Battery [30] attempting to cover the cognitive domains most commonly affected by MS. Moreover, in studies with MS patients [31, 32] the National Adult Reading Test [33, 34] Second Edition was used. The National Adult Reading Test is a test of premorbid intellectual functioning [35]. The Pyramids and Palm Trees Test was created in 1992 by Howard and Patterson [36] to measure the capacity to access detailed semantic information about words and objects and was used by researchers in patients with MS [31, 37].

Furthermore, The Expanded Disability Status Scale (EDSS) [38] is the most widely used instrument for evaluating disability in MS patients [39].

The evaluation of speech and language parameters, particularly verbal learning, has been conducted using various assessment tools in studies with MS patients. One such tool is the Rey Auditory Verbal Learning Test [40, 41]. Furthermore, in 2020 developed the Communication and Language Assessment Questionnaire for persons with Multiple Sclerosis [42], a reliable and valid tool that assesses self-perceived communication and language function in MS [42]. Furthermore, the Addenbrooke’s Cognitive Examination Revised [43] was used in studies for MS patients [44, 45] and contains 5 domains, between them one fluency domain and one language domain [43].

More specifically, to evaluate speech in MS patients, studies used many tools for that scope such as (a) the Assessment of Intelligibility of Dysarthric Speech Sentence Intelligibility Task [46, 47], (b) the speech pathology-specific questionnaire for patients with multiple sclerosis [48, 49], (c) the Dysphonia Severity Index [50], the GRBAS scale [51–53], the Voice Handicap Index, a self-reporting tool [54], the standardized speech tasks [55, 56], the Formant Centralization Ratio [57, 58] has been used as an acoustic metric of dysarthric speech. In terms of tongue control and function in MS a study [59] suggest that the quantitative motor measurement [60] of tongue function might proof useful efficient method to assess motor dysfunction in MS. To assess ataxia in MS patients, the Scale for the Assessment and Rating of Ataxia was developed [25, 61]. Each word is presented individually, and subjects are required to read each aloud [35]. The aforementioned assessment tools and measures contribute to a comprehensive evaluation of speech (voice, motor control) in individuals with MS, providing valuable insights for diagnosis, monitoring, and treatment planning.

This narrative review aimed to identify, enhance, update, and delve into our present comprehension of the type of speech deficits observed in patients with MS and the methodology (assessment tools) that studies followed that were published from May 2015 to June 2022.

## Materials and methods

Α literature search was conducted on the MedLine and Scopus bases in June 2022 with the keywords ‘multiple sclerosis’, ‘speech disorders’, ‘dysarthria’, ‘communication disorders’, ‘phonological disorders’, ‘speech pathology’, ‘anomia’, ‘dysphonia’ and ‘voice problems’. We did not use PRISMA guidelines while conducting our review as it is a narrative review of the existing literature.

The dataset of the current study spanned from May 2015 to June 2022. There were specific eligibility criteria applied for the inclusion of studies in the current review. These were the following: For a study to be included it had to (1) have original data, (2) be conducted on patients with MS who exhibit speech difficulties, (3) focus on speech deficits that patients with MS face, (4) be written and presented in English, and (5) be published from 2015 to 2022. The exclusion criteria were the following: (1) no original data (letters to editor or other reviews were excluded), (2) the study targeted language and cognitive disorders, (3) the study targeted dysphagia, (4) the study targeted treatment methods, and (5) the study was published before 2015. Following database screening, titles and abstracts were reviewed to verify the inclusion criteria. An additional literature search was conducted for related references included in the manuscripts. After duplicates were removed, the suitability of the scanned abstracts was assessed by two independent individuals. Then the full texts were retrieved and read making sure that they met the eligibility criteria applied for this review. Conflicts were resolved after discussion between the authors. Following this, the results of the studies were compiled and presented in two different tables. In the first table, general information and sample characteristics of each study are provided. The second table includes a summary of the results obtained from speech assessments along with the main findings of the studies.

## Results

The data sets of the studies reviewed in this paper were presented according to the following variables: type, mean age of the participants, gender, tests used to establish diagnosis and additional details. With regard to results, all studies used control group of healthy participants. Most of studies reported that EDSS was used to establish diagnosis. Furthermore, almost all studies clearly reported that the patients involved were over 18 years of age except from two studies that had no reference that the patients were over 18 years of age. Summary in the studies 281 males and 628 females recruited. Regarding the exclusion criteria, an acute upper airway respiratory infection, patients to be relapse-free for a month at least prior to testing, voice disorders, larynx malignance, no other neurological disorders other than MS, no vision or hearing problems were required. In addition, the inclusion criteria, patients’ ability to fill in questionnaires, a neurologically confirmed diagnosis of MS, symptoms of dysarthria. More information is shown in Table 1.

**Table 1:** Study information and sample characteristics of the articles included in the review

Study	Dx/type	Mean age	Male	Female	Tests used to establish diagnosis	Additional details
[62]	47 RRMS 20 HC	35,42	19	28	EDSS [38]	Inclusion criteria were the following: the patients had to be monolingual (the primary language of the patients was Persian) Exclusion criteria included the following: 1) an upper respiratory tract infection or colds within the 3 weeks prior to the sample recording, 2) previous vocal problems, 3) laryngeal microsurgery, 4) recent endotracheal intubation, 5) severe problems in speech perception, 6) smoking, 7) drug or alcohol consumption, 8) anatomical problems of articulatory organs (lips, tongue, etc.), 9) hormonal disorders or hormone therapy, and 10) the patient being in a relapse phase within a month preceding the study No evidence that patients’ age was over 18 Control group existence
[63]	107 RRMS 18 SPMS 10 PPMS 6 CIS 70 HC	44	40	101	McDonald Criteria [64], EDSS [38] MSFC [65]	Inclusion criteria: a neurologically diagnosis confirmed All patients were relapse-free for at least 30 days prior to testing The patients’ age was over 18 Control group existence
[31]	100 RRMS 40 HC	40.85	32	68	-	Inclusion criteria were the following: a) definite diagnosis of MS, as confirmed by neurological diagnosis, b) the patients were native English speakers and had enough mobility in the upper limbs to be able to fill in questionnaires The patients’ age was over 18 Control group existence
[66]	7 SPMS 2 RRMS 2 PPMS 14 HC	53.1	6	5	–	Inclusion criteria: a) Only patients with MS who exhibited symptoms of dysarthria as determined by a certified speech-language pathologist with expertise in motor speech disorders were included in the current study b) All participants were required to pass a standard hearing screening and achieve a score of ≥ 25/30 on the Mini Mental Status Examination (MMSE) The patients’ age was over 18 Control group existence
[67]	50 MS 50 HC	48.5	38	62	–	Exclusion criteria included: 1) presence of voice disorder prior to appearance of neurological or clinical symptoms, 2) previous larynx microsurgery, 3) recent episode of endotracheal intubation, 4) primary or metastatic tumor of the larynx, 5) lung or mediastinum, diagnosis of respiratory disease (acute/ chronic) and 6) any other neurological disorders other than MS The patients’ age was over 18 Control group existence
[51]	38 RRMS 38 HC	44	15	23	EDSS [38]	Patients with MS were selected randomly Exclusion criteria included the following: 1) endotracheal intubation within 3 months before entry, 2) history of laryngeal malignancy, 3) an operation performed on the larynx and vocal chords, or an acute upper airway respiratory infection The patients’ age was over 18 Control group existence
[55]	118 MS 22 HC	45.5	30	88	EDSS [38]	Exclusion criteria included the following: 1) presence of other neurological or neuromuscular disorder, 2) MS relapse within the last 3 months, 3) impaired vision or hearing resulting in an inability to complete the testing protocol and 4) speech impairment not related to MS (e.g., stuttering, vocal tics) The patients’ age was over 18 Control group existence
[59]	18 RRMS 11 SPMS 4 PPMS 23 HC	38.8	10	23	EDSS [38]	Inclusion criteria: Participants were MS patients with individual disease burden as expressed by the Expanded Disability Status Scale (EDSS) and with microstructural brain damage as measured by the fractional anisotropy (FA) on Diffusion Tensor Imaging were performed The patients’ age was over 18 Control group existence
[61]	85 MS 21 HC	47.8	22	63	EDSS [38]	Exclusion criteria included the following: [1] the presence of other neurological or neuromuscular disorder, [2] MS relapse within the last 3 months, [3] impaired vision or hearing resulting in an inability to complete the testing protocol and [4] speech impairment not related to MS (e.g., stuttering, vocal tics) The patients’ age was over 18 Control group existence
[68]	18 SPMS 35 RRMS 7 HC	49.5	18	35	–	The statistical analysis shows that both the results from the acoustical analysis and vowel metric analysis have a confidence level of 95% and thus the healthy and non-healthy subjects show significant differences No evidence that the patients’ age was over 18 Control group existence
[69]	97 RRMS 15 SPMS 3 CIS 8 PPMS 60 HS	44	31	92	MRI MSFC [65] EDSS [38]	MS patients had eight years elementary education and no professional use of their voice through employment The patients’ age was over 18 Control group existence
[70]	48 MS with dysarthria 12 MS without dysarthria 12 HC	52	20	40	McDonald Criteria [64]	All participants spoke standard American English and reported no vision or hearing problems or use of a hearing aid, no substance abuse, no other neurological or neuropsychiatric diseases, no use of corticosteroids for the relapse of MS within 8 weeks of testing, and no medication changes for treatment or symptoms of MS within 12 weeks of testing The patients’ age was over 18 Control group existence

*MS* multiple sclerosis, *RRMS* relapsing–remitting multiple sclerosis, *PPMS* primary progressive multiple sclerosis, *SPMS* secondary progressive multiple sclerosis, *HC* healthy control, *CIS* clinically isolated syndrome, *EDSS* Expanded Disability Status Score, *MSFC* Multiple Sclerosis Functional Composite, *MRI* magnetic resonance imaging

For each study under review, the data obtained from the tests of speech assessment along with the results that were drawn, discussed and the main conclusions are presented in Table 2.

**Table 2:** Summary of the aims, speech assessments, and main findings of the studies

Studies	Study Aim	Assessment	Main findings	Conclusions
[62]	To examine the correlation between the characteristics of dysphonia and vowel impairment with disease severity and duration. The specific questions in this study are: [1] Are these subsystems’ changes affected by the disease duration and the disease severity? [2] Are these indexes practical for diagnostic processes of MS? And do they increase the accuracy of diagnosis of speech impairments related to MS?	a) Dysphonia Severity Index Acoustic assessment by calculation of the betascore, by means of measuring four vocal parameters including: maximum phonation time, Jitter percent, highest fundamental frequency, and lowest intensity b) Formant Centralization Ratio by examiner's hand signals, the subjects were asked to read written forms of three words with consonant–vowel-consonant syllable structure	The patients exhibited difficulties: a)Phonation subsystem: loudness control impairment, inability to keep long-term phonation, decrease in habitual pitch, increase in fundamental frequency in MS patients and frequency perturbation in male MS patients, deviations in vocal quality, lower rang of fundamental frequency b) articulation subsystem: a limited range of motion from the articulatory organs, decrease in the vowel space areas, vowel formants shift to the center of the oral cavity	a) Impairments in the phonation system resulting from neurological conditions may in effect be a symptom indicative of MS presence b) Impairments affecting the articulation system along with vowel articulation impairments can be a sign of the progression of the disease c) Phonation subsystem changes not related to the disease severity and the disease duration
[63]	(i) To characterize motor speech disorders in MS including the estimation of prevalence, severity, type and primary manifestations of dysarthria; (ii) to identify relationships between the severity of speech disorder and neurological involvement; and (iii) to examine effect of the pyramidal and cerebellar systems on speech phenotypes	Objective acoustic speech assessment including subtests on phonation, oral diadochokinesis, articulation and prosody was performed	26 patients (18%) showed spastic-ataxic dysarthria, 9 patients (6%) spastic dysarthria, 4 patients (3%) ataxic dysarthria and 1 patient (1%) had non-specific components of dysarthria Speech abnormalities in MS (percentage of sample) were related to monopitch (35%), articulatory decay (26%), excess loudness variations (20%), slow rate (19%), irregular pitch fluctuations (19%), imprecise consonants (15%), slow sequential motion rates (14%), irregular sequential motion rates (13%), increased noise (13%) and signal perturbations (8%)	Patients with MS developed mainly mild spastic-ataxic dysarthria. They experienced difficulties in all investigated components of speech production including phonation, oral diadochokinesis, articulation and prosody. However, prosodic- articulatory disorder was the most salient with manifestations such as: monopitch, articulatory decay, excess loudness variations and slow rate
[31]	To investigate the extent and nature of anomic symptoms in people with Rapidly Evolving Severe Relapsing–Remitting Multiple Sclerosis with respect to both accuracy and reaction time in a picture naming assessment task	a) The Addenbrooke’s Cognitive Examination–Revised (Mioshi et al., 2006) tested by asking the patient. b) The National Adult Reading Test (Nelson & Willison, 1991) c)The Pyramids and Palm Trees (Howard & Patterson, 1992) tested by using either pictures, or written or spoken words to change the modality of stimulus or response items	The patients showed slow latencies, weak phonation, low voice volume and reduced articulatory precision in production of multi-syllabic words	No evidence of severe dysarthria in reading single words
[66]	To identify tongue, lip, and jaw motor deficits in persons with dysarthria due to MS to better understand the speech motor mechanisms that underlie their aberrant speech	A standard speech evaluation was administered during the study to obtain speech severity ratings, speech intelligibility scores, and speech perceptual characteristics. Specifically, sentence intelligibility scores were calculated based on a standardized reading test consisting of eleven sentences that vary in length (Sentence Intelligibility Test)	The patients in the study exhibited longer movement durations, reduced tongue peak speeds, reduced peak speed/displacement ratios of the lower lips and jaw particularly during the closing gesture	The authors suggested that their findings indicate that speech treatments should specifically target tongue speed when aiming to reduce speech unnaturalness
[67]	Αimed at measuring maximum phonation times, maximum expiratory times, and articulation abilities scores in patients with MS compared to healthy subjects and at investigating if any of these parameters could be used as a measure of MS progression	a) Articulation subtest from the Fussi assessment (Dysarthria scores) b) EDSS	a) MS patients had reduced maximum expiratory times and reduced maximum phonation times as compared to healthy controls b) Articulation scores in MS patients were abnormal c)The expiratory times are positively correlated with the maximum phonation times, and the latter are negatively correlated with the articulation scores. The EDSS scores are negatively correlated with the maximum expiratory times Based on this correlation the authors proposed the use of maximum expiratory times to monitor MS progression	As the expiratory times were significantly correlated with the EDSS scores, they could be used to measure the severity of MS and to monitor its progression
[51]	To compare the results of voice self-assessment with the results of expert perceptual assessment in patients with MS	a) Voice Handicap Index, a standardized 30-point questionnaire (Jackobson et al., 1997). b) GRBAS scale according to the Japanese Society of Logopedics and Phoniatrics c) EDSS	The patients in this study presented intense vocal difficulties Symptoms of dysphonia ranged from 30 to 70%	A significant number of patients with MS experienced voice problems. The Voice Handicap Index is a good and effective tool to assess patient self-perception of voice quality, but it may not reflect the severity of dysphonia as perceived by voice and speech professionals The authors concluded that almost half of the MS patients feel and describe mild dysphonic difficulties which in turn affect the quality of their lives
[55]	The primary aim of the current study was to describe the relationship between speech measurements and general neurological impairment, brain volume, brain lesion load and quality of life in MS in a single cohort. The secondary aims were to determine the association between acoustic metrics and neurological dysfunction in non-dysarthric pwMS, and to estimate at which level of neurological disability each speech metric changes	EDSS scores Analysis of speech variables through perceptual and acoustic methods. Elicited speech using five standardized speech tasks that fit along a spectrum of automaticity, from simple to complex (phonetically and/or cognitively), including i) vowel, ii) DDK, iii) reading and iv) unscripted monologue	Dysarthria frequency and severity were associated with EDSS. The three characteristics with the strongest associations with EDSS were the same for perceptual and acoustic analysis and included DDK rate, speech rate and increase in pauses/intervals. DDK rate and speech rate were affected mainly in the severe group, whereas the increase in pauses was observed for both the moderate and severe groups The patients exhibited slower speech rate, increased variation in speech rate, increase in pauses and smaller pitch variation (or monotonic speech) during connected speech as this was evident at least for the majority of MS patients in comparison to controls	Both the acoustic composite score and perceptual naturalness, global measurements of speech function, were associated with EDSS-defined disease severity. The degree of speech impairment moderately parallels that of overall MS-related neurological impairment and was not restricted only to the severe phases of MS
[59]	Aimed to explore the association of tongue motor dysfunction in MS patients with overall clinical disability and structural brain damage	Employed a force transducer based quantitative-motor system to objectively assess tongue function. The TVF output and the mean applied tongue force were measured during an isometric tongue protrusion task	MS patients showed significantly increased tongue force and decreased tongue force compared to controls. TFV but not TF was correlated with the EDSS	TFV might serve as an objective and non-invasive outcome measure to augment the quantitative assessment of motor dysfunction in MS
[61]	Aimed to build an objectively measured speech score that reflects cerebellar function, pathology and quality of life in MS	a) Analysis of speech variables through perceptual and acoustic methods b) Scale for the assessment and rating of ataxia (Schmitz-Hu¨ bsch, 2006)	In this study it was noted that the degree of cerebellar dysfunction as this was measured by SARA, was associated mainly with impaired naturalness and intelligibility, slow speech rate, prolonged pauses and inaccurate pronunciation of vowels and consonants	Speech measurements such as an increase in pauses, slower maximum speech rate and subclinical voice tremor are associated with cerebellar dysfunction in MS. When combined, these measures are highly predictive of cerebellar dysfunction in MS
[68]	To propose a method for the evaluation and identification of significant patterns in voice samples acquired from patients affected by MS. This could enhance early detection, differential diagnosis and monitoring of disease progression	Acoustic analysis of parameters F0; Jitter; Shimmer; Harmonic to Noise Ratio and vowel metric, Vowel Space Area to evaluate the vowel articulation) have been used to compare the vocal signals of healthy subjects and the MS affected patients	Acoustic Analysis: a) The results show an increase of the F0 average in men for SPMS; instead, F0 average decreases in women both for Secondary SPMS and RRMS. b) Showed great differences between healthy subjects and MS patients, with the parameters of relative jitter and relative shimmer to constitute indicators of MS disease Vowel analysis: The vowel area decreases for pathological subjects. Remarkable reduction of the area for patients with RRMS, even though a tVSA increases can be notified in SPMS	The results showed different values for all parameters, distinguishing normal and pathological subjects as indicated in the literature. The procedure is appropriate to be used in early diagnosis that is critical in order to improve the passions quality of life
[69]	To explore relationships between spastic and ataxic dysarthria patterns evaluated using acoustic analyses and different MRI markers of whole and regional brain atrophy. Specifically, hypothesized that aspects of ataxic dysarthria such as irregular pitch variability, irregular oral diadochokinesis, and excess loudness variations would reflect cerebellar white and grey matter loss, whereas slowness of speech associated with spastic dysarthria would be related to total white and cortical grey matter loss due to widespread brain atrophy	Acoustic speech assessment included: a) Pitch variability, b) Diadochokinetic rate, c) Diadochokinetic regularity, d) Articulation rate, e) Loudness variability	A link of brain atrophy with a) slower articulation during reading and with b) a reduced diadochokinetic rate during rapid syllable repetition	Even though the articulation rate is not generally a comprehensive measure in MS, the authors found a strong correlation between brain atrophy and articulation rate, while EDSS and MSFC measurements provided similar results. According to the authors slow articulation rate during reading was particularly associated with bilateral white and grey matter loss while reduced maximum speed during oral diadochokinesis was a result of greater cerebellar involvement
[70]	To investigate the impact of cognitive impairment on speech produced by speakers with MS with and without dysarthria	a) Speakers produced a spontaneous speech sample to obtain speech timing measures of speech rate, articulation rate, and silent pause frequency and duration b) Twenty listeners judged the overall perceptual severity of the samples using a visual analog scale that ranged from no impairment to severe impairment (speech severity)	a) Speech timing was significantly slower for speakers with dysarthria compared to speakers with MS without dysarthria. b) Silent pause durations also significantly differed for speakers with both dysarthria and cognitive impairment compared to MS speakers without either impairment c) Significant interactions between dysarthria and cognitive factors revealed comorbid dysarthria and cognitive impairment contributed to slowed speech rates in MS, whereas dysarthria alone impacted perceptual judgments of speech severity. d) Speech severity was strongly related to pause duration	The findings suggest the nature in which dysarthria and cognitive symptoms manifest in objective, acoustic measures of speech timing and perceptual judgments of severity is complex

*MS* multiple sclerosis, *DDK* diadochokinetic, *EDSS* Expanded Disability Status Scale, *TVF* Tongue force variability, *SPMS* secondary progressive multiple sclerosis, *RRMS* relapsing–remitting multiple sclerosis, *F0* fundamental frequency of a speech signal, *tVSA* triangular vowel space area, *MRI* magnetic resonance imaging, *MSFC* Multiple Sclerosis Functional Composite

Most of studies referred about the effects of MS on motor movement are which includes muscle weakness and what is the reason for muscle weakness, spasticity and loss of coordination and what are the symptoms of MS. As a result, the motor system is impaired and MS patients in their majority also deal with different forms of dysarthria, although not all studies have reached the same conclusion. Dysarthria is considered the primary cause of communication deficit in MS, yet patients with MS present with concurrent cognitive deficits that can interfere with effective communication.

Moreover, the articulation system is impaired. The studies indicated that MS affects the articulators per se and consequently patients’ speech rate. Articulation was analyzed across the studies and was characterized by consonant imprecision, decreased word output rate and slow vowel transitions likely due to slow tongue movements. Findings recognized that MS is a condition that can negatively affect the phonation system. MS patients face dysphonic problems, although there remains to define the severity level and its correlation to other factors. Voice quality was studied in studies [51, 62]. The studies that encompassed the documentation of perception primarily adopted a descriptive approach. The majority of multiple sclerosis patients were evaluated as having vocal impairments by speech pathologists. However, respiratory issues and voice impairments were comparatively given less attention and were not thoroughly examined. Furthermore, studies reported on the presence of prosody in patients [51, 63]. The speech rate of patients with MS was observed to be reduced and slower, as evidenced by a decrease in the number of syllables per second and a lower production of words per minute when compared to healthy control groups. In relation to tasks involving reading and speech, it was observed through acoustic analysis that individuals with multiple sclerosis exhibited a greater frequency and duration of pauses.

With regard to the main outcome of the studies, dysarthria seems to be a common symptom in MS patients in most of the studies as well as in two studies [62, 69]. In the studies [62, 68] there was an evaluation of acoustic analysis along with a vowel metric analysis to evaluate speech features and identify significant patterns in voice samples of patients with MS [68]. The authors considered to better define disordered voices against healthy ones, acoustic analytic approaches have been suggested for implementation of this procedure. In one study [70] problems with speech extraction were identified. Speech timing was significantly slower for MS patients with dysarthria compared to MS patients without dysarthria. In addition, silent pause durations also significantly differed for MS patients with both dysarthria and cognitive impairment compared to MS patients without either impairment [70]. Furthermore, in other studies [55, 67], Expanded Disability Status Score outcomes were used in support of the general idea that speech impairment is strongly correlated to the level of MS severity. However, in this study [51] due to the limited number of subjects and due to the limited parameters, that they investigated could not draw any solid conclusions about the level of severity of dysphonia that MS patients exhibited.

Evidence from the studies [55, 61] suggests that MS patients demonstrate abnormalities in their speech rate. Specifically, the studies revealed that the pace of speech was comparatively reduced and there was a rise in its variability. Furthermore, it was observed that individuals with multiple sclerosis exhibited an augmentation and extension of the pauses present in their speech. The amplitude of the vocal inflections exhibited a reduced variance, resulting in a uniform and unvarying quality of speech. Cerebellar dysfunction was found to be associated with subclinical voice and tremors.

Finally, the pronunciation of vowels and consonants was inaccurate. A study [69] found that MS patients exhibited slow articulation during reading and reduced sequence rhythm during rapid syllable repetition, as tests for joint rhythm, auditory speech and sequence rhythm indicated. Furthermore, 2 studies [59, 66] addressed the fact that there are motor problems, trembling and problems with the joints in MS patients. It was found that there were reduced ratios of maximal velocity displacement of the lower lips and jaw reduced peak velocities of the tongue. Based on these results it was pointed out that the ability to move the tongue with adequate speed during speech was significantly impaired in patients with MS, providing thus an explanation for their slowed speech rate. Therefore, it was suggested that compensatory strategies are in need during speech treatment in order to ‘maximize speech clarity in the presence of the impaired tongue motor performance’.

## Discussion

The aim of the current literature review was to identify, record, discuss, and delve into the type of speech deficits observed in patients with MS. MS patients can exhibit a range of symptoms in various domains including speech as well. From the aforementioned studies, it is apparent that MS patients face difficulties concerning a number of components of speech including phonation, oral diadochokinesis, articulation, and prosody. These findings have been previously reported in the MS literature and it seems that they are prominent in the majority of patients. Considering the speech-related findings of the studies reviewed above, one of the most commonly identified symptoms of MS was articulation difficulties. In some of the studies reviewed the patients included, exhibited dysarthria [63, 68, 70], while in another study, actual cases of dysphonia were reported [51]. In other studies, it was also noted that MS patients also face a range of deficiencies regarding speech articulators per se as a result of an impaired motor system [59, 66]. However, more research needs to be carried out in order to further address the issue of the way that these deficiencies affect the articulation system of MS patients.

The second most reported deficit in MS patients was impaired phonation and a slow speech rate [51]. These findings are in line with the MS literature [71] according to which phonation difficulties such as vocal deficits, breathiness, volume abnormalities, etc., are present in many MS patients. Slow speech rate and long/extended pauses are also commonly observed and described in MS patients [69, 70]. Even though respiratory problems and resonatory impairments were less commonly described than other deficits [67], they still significantly impact patients' everyday lives. However, studies investigating these aspects are scarce, and because they do not follow a standard methodology, no solid conclusions can be drawn. The objective of this review is to enhance and revise our present comprehension of dysarthria in individuals with multiple sclerosis (PwMS),

## Conclusion

The current literature review aimed to enhance, update, and delve into our present comprehension of a) the type of speech deficits observed in patients with MS, and b) the methodology (assessment tools) studies followed. In the literature on MS, speech difficulties are notable among patients due to an impairment of the motor system and its underlying anatomical structures. Nonetheless, the main bulk of studies indicates that patients with MS develop dysarthric characteristics. It is important for speech and language therapists working with MS patients to be aware of possible cognitive-linguistic impairments and take this into account when assessing, managing, and intervening. Taking in mind these, there is a need for more studies to be conducted that will apply a more systematic methodological approach and similar inclusion criteria to categorize various speech manifestations better and enhance our understanding of the patterns that may be (or not) associated with the specific clinical subtypes of MS. By doing so better intervention and treatment methods can be discovered and applied that will improve the communicational function, the psychological well-being, and the quality of MS patients’ life.

## Future directions

Finally, it would be interesting for future research to systematically investigate possible correlations between the different clinical types of MS and speech deficits. It is essential, though, to stress that to achieve common ground among other studies, similar methodologies and inclusion criteria should be applied. To this end, it is evident that more research must be carried out about the etiology of MS, the neuroanatomical correlates, along with the definition of clinical, cognitive, and linguistic patterns present in each phase to develop better methods of intervention and treatment of MS patients and improve their communication ability and quality of life.

## Data Availability

Not applicable.
